# Development and Validation of a Risk Score for Predicting Non-Adherence to Antiretroviral Therapy

**DOI:** 10.1089/aid.2022.0147

**Published:** 2023-10-03

**Authors:** Pablo Acin, Sonia Luque, Isaac Subirana, Joan Vila, Xènia Fernández-Sala, Ana Guelar, Marta de Antonio-Cuscó, Itziar Arrieta, Hernando Knobel

**Affiliations:** ^1^ Pharmacy Service Colisée Barcelona Isabel Roig, Barcelona, Spain.; ^2^ Pharmacy Service Hospital del Mar, Infectious Pathology and Antimicrobials Research Group, Institut Hospital del Mar d'Investigacions Mèdiques (IMIM), Universitat Autònoma de Barcelona, Barcelona, Spain.; ^3^ Centro de Investigación Biomédica en Red de Enfermedades Cardiovasculares (CIBERCV), Institut Hospital del Mar d'Investigacions Mèdiques (IMIM), Barcelona, Spain.; ^4^ Pharmacy Service Hospital del Mar, Barcelona, Spain.; ^5^ Department of Medicine, Infectious Diseases Service Hospital del Mar, Barcelona, Spain.; ^6^ Infectious Diseases Service Hospital del Mar, Barcelona, Spain.; ^7^ XECS-Universitat Pompeu Fabra Barcelona, Barcelona, Spain.; ^8^ CIBER of Infectious Diseases (CIBERINFEC CB21/13/00002), Institute of Health Carlos III, Madrid, Spain.

**Keywords:** adherence, antiretroviral therapy, risk factors, score, validation

## Abstract

Several patient-related factors that influence adherence to antiretroviral therapy (ART) have been described. However, studies that propose a practical and simple tool to predict nonadherence after ART initiation are still scarce. In this study, we develop and validate a score to predict the risk of nonadherence in people starting ART. The model/score was developed and validated using a cohort of people living with HIV starting ART at the Hospital del Mar, Barcelona, between 2012 and 2015 (derivation cohort) and between 2016 and 2018 (validation cohort),. Adherence was evaluated every 2 months using both pharmacy refills and patient self-reports. Nonadherence was defined as taking <90% of the prescribed dose and/or ART interruption for more than 1 week. Predictive factors for nonadherence were identified by logistic regression. Beta coefficients were used to develop a predictive score. Optimal cutoffs were identified using the bootstrapping methodology, and performance was evaluated with the C statistic. Our study is based on 574 patients: 349 in the derivation cohort and 225 in the validation cohort. A total of 104 patients (29.8%) of the derivation cohort were nonadherent. Nonadherence predictors were patient prejudgment; previous medical appointment failures; cultural and/or idiomatic barriers; heavy alcohol use; substance abuse; unstable housing; and severe mental illness. The cutoff point (receiver operating characteristic curve) for nonadherence was 26.3 (sensitivity 0.87 and specificity 0.86). The C statistic (95% confidence interval) was 0.91 (0.87–0.94). These results were consistent with those predicted by the score in the validation cohort. This easy-to-use, highly sensitive, and specific tool could be easily used to identify patients at highest risk for nonadherence, thus allowing resource optimization and achieving optimal treatment goals.

## Introduction

Nonadherence to antiretroviral therapy (ART) is the greatest predictor of virologic failure and emergence of HIV-1 drug resistance and is highly correlated with morbidity and mortality in people living with HIV (PLHIV).^1^ Despite this established link between nonadherence and negative treatment outcome, a meta-analysis, including 84 studies, found that only 62% of PLHIV on ART achieved optimal adherence. This meta-analysis aimed to determine the mean proportion of people reporting ≥90% adherence to a prescribed ART.^2^

The different factors that may interfere with ART adherence have been widely described over the years. Suboptimal adherence has been strongly associated with patient beliefs about the treatment, including concerns about adverse effects or even the necessity of being treated,^3^ recreational drug abuse,^4^ alcohol use,^5^ mental diseases,^6^ lower income and education,^7^ bad nutrition,^8^ poor social support,^7^ or bad accessibility and long distance from the health care center.^9^

Recent studies have expanded the focus of adherence to include new aspects such as retention in HIV medical care, which includes attending regular clinical follow-up visits,^10^ and the stigma that accompanies PLHIV, which further increases the risk of poor ART adherence.^11^ The multiplicity and frequency of all of these factors highlight the need for identifying high-risk patients to allow development of efficient strategies for adherence improvement, such as simplifying ART or providing better adherence education and advice as well as closer follow-up.^3,12^

To date, there are few available tools, including the aforementioned risk factors, to efficiently predict ART adherence.^13–16^ Moreover, the existing models have been developed based on specific populations and often use varying adherence definitions or measurements and, furthermore, include different predictive variables. This heterogeneity makes it difficult to widely apply these models for ART adherence prediction.

The aim of this study was to develop and validate a score, including the main nonadherence risk factors described in the literature, to predict the risk of nonadherence in PLHIV, which can be easily used in daily clinical practice.

## Patients and Methods

### Study population

A retrospective observational study was performed at the Hospital del Mar, a 400-bed university tertiary hospital in Barcelona, Spain. The cohort for determining predictive parameters included 349 PLHIV, aged >18 years, who initiated ART between 2012 and 2015. Data from 225 PLHIV who started ART between January 2016 and February 2018 were used for validation. The starting point of follow-up for PLHIV was their ART initiation and the endpoint for all of them was February 2019. PLHIV from both cohorts were followed up for at least 1 year after ART initiation.

The inclusion criterion for the derivation and validation cohorts was ART naivety or ART restarted after a previous episode of treatment interruption for longer than 6 months. The exclusion criterion was missing data (incomplete medical records).

### Data collection and identification of risk factors for nonadherence

We collected the following patient data: demographics, epidemiological data, cultural and social characteristics, cause of HIV infection, baseline CD4 lymphocyte count, and plasma HIV-1 RNA viral load. Data regarding ART included active agent and number of tablets per day. Data were both collected during the first medical visit and extracted afterward from medical records and electronic prescriptions. To assess ART efficacy, the plasma HIV-1 RNA viral load was recorded after at least 1 year of treatment follow-up.

The patient-related potential risk factors for nonadherence were chosen according to those described in previous studies,^3–7^ as follows: (i) heavy alcohol use: >14 drinks/week or >4 drinks/day (National Institute on Alcohol Abuse and Alcoholism definition)^5^; (ii) substance abuse: active cocaine use (including crack) and/or active heroin use^17^; (iii) cultural and/or idiomatic barrier: requires *a translator* or insufficient educational/cultural level to understand medical explanations^18^; (iv) unstable housing: includes individuals who lack a permanent residence, such as those who are homeless and living on the street or in shelters; individuals who face housing challenges, such as struggling to pay rent, experiencing overcrowding, moving frequently, or spending a significant portion of their household income on housing; and individuals who are socioeconomically vulnerable and included in social programs^19^; (v) prejudgment by patient: patient clearly expressed denial of the existence of HIV and the effectiveness of the treatment and/or the health system; (vi) previous appointment failures: at least two previously missed medical appointments before the treatment commenced; and (vii) severe mental illness: any clinical diagnosis of bipolar disorder, chronic major depressive disorder with psychotic features, schizophrenia, schizoaffective disorder, and other nonsubstance abuse-related psychoses, as diagnosed by a psychiatrist.^20^

### ART adherence measurement

ART adherence was assessed by pharmacy refill every 2 months. This information was complemented by adherence based on patient self-reports during medical appointments with clinicians and pharmacists.

Adherence was quantified by the medication possession ratio (MPR), which was defined as the sum of daily doses for all drugs in a particular time period divided by the number of days in the same period, and expressed as percentage.

### Nonadherence

Nonadherence to ART was defined as <90% adherence of the prescribed dose (MPR <90%) and/or ART interruption for more than 1 week. PLHIV lost to follow-up (LTFU) for unknown reasons (not being transferred to another hospital) were also considered as nonadherents.

### Clinical outcomes

Treatment failure was defined as two consecutive plasma HIV-1 RNA viral loads >50 copies/mL, death, or loss to follow-up for unknown reasons. PLHIV lost to follow-up due to transfer to another hospital or imprisonment were not considered as treatment failure.

### Statistical analyses

Categorical variables are presented as percentages and continuous variables as median and quartile 1–quartile 3 [interquartile range (IQR)] values or mean [±standard deviation (SD)]. Risk factors are presented as dichotomous variables. Continuous variables were compared using Student's *t*-test for those with a normal distribution and using the nonparametric Mann–Whitney *U* test when normality could not be assumed. For dichotomous variables, the chi-square and Fisher exact tests were applied.

Logistic regression was used to explore various risk factors associated with nonadherence. Collinearity issues were tested using the variance inflation factor (VIF) as a measure. Univariate analyses were performed separately for each of the risk factor variables to ascertain the odds ratio (OR) and 95% confidence interval (CI). Variables with a *p* value < .2 in the univariate analyses were included in the logistic regression model for the multivariate analysis. A backward selection process was used.

Statistically significant risk factors (*p* < .1), in the multivariate analysis, were retained in the model. Results of the logistic regression analysis are reported as adjusted ORs with 95% CIs. Model discrimination capacity and calibration were assessed using area under receiver operating characteristic (ROC) curve and calibration plots, respectively. Bootstrapping was used to obtain a cutoff that gave an optimal trade-off between sensitivity and specificity and a maximum area under the curve based on the ROC curve.

To facilitate the use of the predictive scale, a weighted average score proportional to regression coefficient β-values was calculated for each risk factor and included into the final predictor model for nonadherence. The C statistic was assessed as a measure of performance.

The nonadherence risk score was validated in the second cohort by assigning the same points to variables as for the derivation cohort. The same cutoffs were used to define high and low risk of nonadherence.

All tests of significance were two-sided, with *p* < .05 as the threshold for statistical significance. The statistical analysis was performed using IBM SPSS Statistics for Windows, Version 23.0 (IMB Corp., Armonk, NY).

### Score calculator

The developed nonadherence risk predictive score is available on the website of the Service of Infectious Diseases, Hospital del Mar (http://artshiv-calculator.humimar.org/en).

Appendix Table A1 shows an example of total score and nonadherence probability for two subjects.

### Ethics statement

The protocol was approved by the Clinical Research Ethics Committee of the “Parc de Salut Mar” (2017/7362/I). Written informed consent was not considered necessary as it was an observational and retrospective study. All patient data were anonymized for the purposes of this study. The confidential information of patients was protected according to the national normative system.

## Results

The derivation cohort included 349 PLHIV with a mean (±SD) age of 39 ± 9.9 years and the majority (85.1%) were male. Thirty-five PLHIV (10.1%) had had a previous ART interruption episode. The median (IQR) follow-up period was 21.4 (10.8–32.8) months and during this time, 70.2% of the PLHIV were considered adherent (*n* = 245) and 29.8% (*n* = 104) were considered nonadherent. In the nonadherent group, 48 PLHIV (19.6%) were lost to follow-up for unknown reasons.

At the end of the study follow-up period, optimal ART efficacy (plasma HIV-1 RNA viral load <50 copies/mL) was achieved in 235 PLHIV (95.9%) in the adherent group and 63 PLHIV (60.6%) in the nonadherent group (*p* < .001). Seventeen PLHIV (6.9%) and 69 PLHIV (66.3%) presented treatment failure during the study follow-up in the adherent group and nonadherent group, respectively (*p* < .001). One patient died in the nonadherent group due to an unknown cause (loss to follow-up).

The validation cohort included 225 PLHIV with a mean (±SD) age of 40 ± 9.7 years and 191 (84.9%) were male. Twenty-nine PLHIV (12.8%) had had a previous ART interruption episode before study inclusion. The median (IQR) follow-up period was 20.0 (11.80–27.90) months, during which 65.3% (*n* = 147) of PLHIV were adherent and 34.7% (*n* = 78) nonadherent. In the nonadherent group, the number of PLHIV lost to follow-up for unknown reasons was 34 (43.6%).

In the adherent group, 142 PLHIV (96.6%) achieved a plasma HIV-1 RNA viral load of <50 copies/mL versus 46 PLHIV (59.0%) in the nonadherent group (*p* < .001). Seven PLHIV (4.8%) presented treatment failure in the adherent group versus 46 PLHIV (59.0%) in the nonadherent group (*p* < .001). Three PLHIV died during the follow-up period due to an unknown cause, all of them in the nonadherent group. No PLHIV were excluded in both cohorts. Baseline characteristics of both cohorts are reported in Table 1.

**Table 1.** Baseline Characteristics of Patients in the Derivation Cohort and Validation Cohort

Characteristic	Derivation cohort (*n* = 349)	Validation cohort (*n* = 225)	*p*
Age, mean (SD)	39.38 (9.86)	39.56 (9.68)	.80
HIV-RNA, log_10_/mL, median (IQR)	4.82 (4.25–5.22)	4.76 (4.20–5.12)	.30
CD4 cell count, cells/mL, median (IQR)	331 (160–476)	382 (166–602)	.13
Months of follow-up, median (IQR)	21.43 (10.80–31.97)	20.00 (11.80–27.90)	.07
Male, *N* (%)	297 (85.1)	191 (84.9)	.69
Nonadherents, *N* (%)	104 (29.8)	78 (34.7)	.22
HIV acquisition through IDU, *N* (%)	93 (26.6)	49 (21.8)	.19
STR, *N* (%)	175 (50.1)	188 (83.6)	<.001
Integrase inhibitor-based ART, *N* (%)	93 (26.6)	172 (76.4)	<.001

ART, antiretroviral therapy; IDU, injection drug use; IQR, interquartile range; SD, standard deviation; STR, single-tablet regimen.

The frequency of all predictive risk factors for nonadherence was higher in the nonadherent group. Previous appointment failures, unstable housing, and substance abuse were factors with the highest prevalence. Table 2 presents the risk factors by adherence group for the derivation and validation cohorts (univariate analysis).

**Table 2.** Risk Factors by Adherence Group in the Derivation Cohort and Validation Cohort

	Derivation cohort				Validation cohort			
	All patients,* N* = 349	Adherent,* N* = 245	Nonadherent,* N* = 104	*p*	All patients,* N* = 225	Adherent,* N* = 147	Nonadherent,* N* = 78	*p*
Alcohol use, *N* (%)	53 (15.2)	15 (6.1)	38 (36.5)	<.001	37 (16.4)	12 (8.2)	25 (32.1)	<.001
Substance abuse, *N* (%)	81 (23.2)	30 (12.2)	51 (49.0)	<.001	83 (36.9)	36 (24.5)	47 (60.3)	<.001
Cultural or idiomatic barrier, *N* (%)	37 (10.6)	10 (4.1)	27 (26.0)	<.001	13 (5.8)	4 (2.7)	9 (11.5)	.01
Unstable housing, *N* (%)	75 (21.5)	22 (8.98)	53 (51.0)	<.001	40 (17.8)	6 (4.1)	34 (43.6)	<.001
Prejudgment by patient, *N* (%)	13 (3.7)	2 (0.82)	11 (10.6)	<.001	12 (5.3)	3 (2)	9 (11.5)	.004
Previous appointment failures, *N* (%)	61 (17.5)	10 (4.08)	51 (49.0)	<.001	54 (24)	12 (8.2)	42 (53.8)	<.001
Severe mental illness, *N* (%)	61 (17.5)	23 (9.39)	38 (36.5)	<.001	40 (17.8)	14 (9.5)	26 (33.3)	<.001

Table 3 shows results of the logistic regression of risk factors independently associated with nonadherence in the derivation cohort. The final score for each risk predictive factor is shown in the column “Score (over 100).” In the collinearity test, in no case did the VIF reach the value of 5 and therefore there were no collinearity problems between variables.

**Table 3.** Multiple Logistic Regression Coefficients for Nonadherent Participants

	Beta coefficient	Score (over 100)	Odds ratio	95% Confidence interval	*p*
(Intercept)	−2.739	—	—	—	—
Alcohol use	1.710	15	5.53	2.44–12.55	<.001
Substance abuse	0.899	8	2.46	1.16–5.21	.019
Cultural or idiomatic barrier	2.007	18	7.44	2.78–19.95	<.001
Unstable housing	1.135	10	3.11	1.43–6.77	.004
Prejudgment by patient	2.402	21	11.05	1.81–67.53	.009
Previous appointment failures	2.352	21	10.50	4.36–25.31	<.001
Severe mental illness	0.743	7	2.10	0.96–4.61	.064

**FIG. 1.**
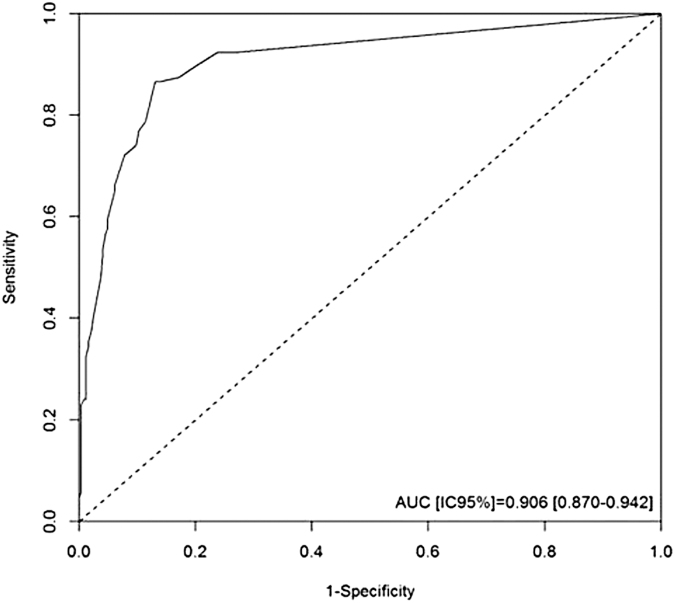
ROC curve of the best cutoff point according to the maximum likelihood criterion: 26.3. ROC, receiver operating characteristic.

To test the validity of the score, all risk scores for each patient were calculated individually, taking into consideration their risk factors. In the ROC curve, the cutoff point with the best specificity and sensitivity to identify a nonadherent patient was 26.3. This threshold exhibited a sensitivity of 0.87 (0.78–0.92), specificity of 0.86 (0.81–0.90), positive predictive value of 0.73 (0.64–0.80), and negative predictive value of 0.94 (0.90–0.96). The C statistic was 0.91 (95% CI 0.87–0.94) (*p* < .001) (Fig. 1).

In the validation cohort, a predefined cutoff point of 26.3 was used to identify patients at high risk for nonadherence. With this threshold, the sensitivity and specificity to predict the real lack of adherence were 0.87 (0.78–0.94) and 0.86 (0.80–0.91), respectively. The positive and negative predictive values were 0.77 (0.67–0.85) and 0.93 (0.87–0.99), respectively. The C statistic was 0.91 (95% CI 0.86–0.95).

**FIG. 2.**
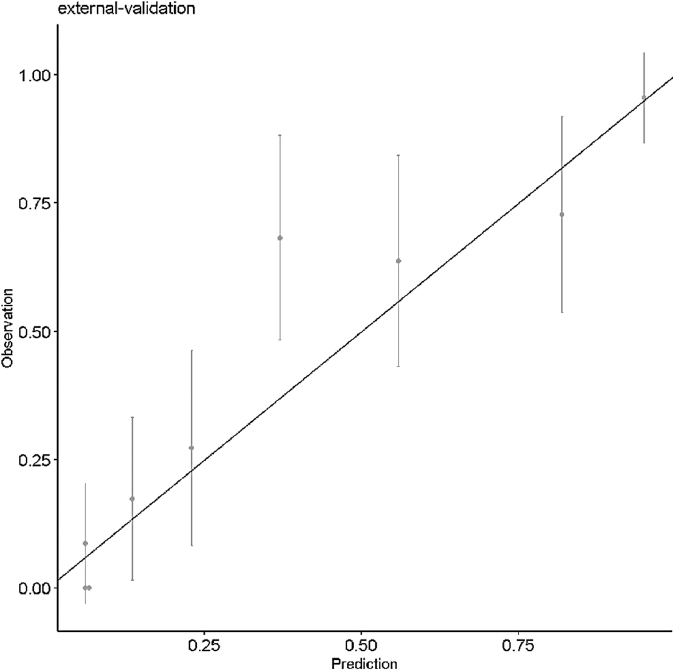
Calibration plot containing 95% confidence intervals of the observed (Y-axis) and model-based (X-axis) nonadherence risk computed for the validation cohort.

Figure 2 shows that the model achieved good calibration in the validation cohort since all intervals except one included the diagonal, indicating good agreement between expected (obtained by the model) and actual nonadherence risk.

## Discussion

We have developed and validated a score for predicting nonadherence to ART. This is the first risk score developed and validated in a Spanish cohort, including several patient factors that are easily collected at the point of care.

Key findings of the study are (i) this tool could help to identify PLHIV at high risk for nonadherence to ART, with high sensitivity (87%) and specificity (86%) and a high negative predictive value (93%); and (ii) the predictive factor that showed the highest influence on nonadherence to ART was prejudgment by the patient about the disease, the treatment, or the health system, consistent with results reported by a meta-analysis conducted in 2016 among patients with long-standing diseases.^21^

In the univariate analysis, severe mental illness was an important factor for predicting nonadherence. Mental problems are strongly associated with other factors, such as *heavy* alcohol use and substance abuse, due to which collinearity exists between both variables. However, both showed a nonsignificant trend in the final model.

Indeed, previous studies have demonstrated the influence of certain psychiatric disorders such as depression^22^ or post-traumatic stress^23^ on adherence to treatment of certain diseases, especially HIV^2,24^ where the prevalence of psychiatric disorders is particularly high.^25^ In our study, we considered severe mental illness as a recorded diagnosis by a psychiatrist, but it is possible that some of our patients presented with *psychiatric problems* that were not diagnosed, a factor that could lead to underestimation of the prevalence in our study.

Two well-defined patient risk factors for nonadherence described in the literature were not included in the study: first, long distance from the health center^9^ was excluded because this problem is common in low-income countries, but not in our environment; and second, this study pays no heed to the economic restrictions on access to treatment since the Spanish health system exempts ART patients from payment. In other countries, such as the United States, payment for these treatments depends on the patient's insurance and financial aspects represent an important barrier to ART compliance.^10^

Table 2 shows relevant differences between the derivation cohort and validation cohort in substance abusers and idiomatic barriers: 37% versus 23% and 6% versus 11%, respectively, and the proportion of people LTFU is higher in the validation cohort. These differences could be explained by changes in referrals of patients by city centers for drug users and the changing migratory processes over time.

One limitation of our study is the fact that only patient risk factors for nonadherence were considered. Other risk factors related to treatment such as pill count, side effect profile, or food requirement^3,13^ were excluded because the goal of the study was to develop an easy to use tool for identifying patients at risk for nonadherence.

The importance of this statement is reflected in Table 1: in the derivation cohort, single-tablet regimen (STR) and integrase strand transfer inhibitors (INSTI) were 50.1% and 26.6%, respectively, while in the validation cohort, STR was 83.6% (*p* < .001) and INSTI was 76.4% (*p* < .001). Nevertheless, adherence results were similar in both cohorts, showing that patient-associated risk factors have a greater influence than treatment-associated factors. Finally, although it is true that MPRs are not exactly the same as adherence, pill-taking behavior is usually correlated with measures of adherence.

Compared with other predictive models of nonadherence, this study has several strengths. First, the developed score can calculate the probability of nonadherence to ART for each individual after considering different patient factors that can be easily collected at the point of care. Another instrument, the Adherence Barriers Questionnaire for HIV (ABQ-HIV), bases risk predictions on 15 psychometric tests that patients have to complete.^13^

Our score requires little patient collaboration and bases the calculation on patient risk factors. This is an advantage as it allows faster risk calculation and, consequently, faster preventive or corrective action. Additionally, in contrast to the ABQ-HIV scale, our 7-item scale includes nonadherence risk factors such as *heavy* alcohol and substance use or social dystopia, which are very prevalent among HIV patients.

Another study that excluded most of these variables was developed by Cheng et al., who identified being African American as the main predictive factor for poor adherence to ART in the United States.^14^ One study similar to our own reported on a probability model for predicting short-term interruptions of ART developed and tested in Uganda.^15^ Some sociodemographic measures are the same in both articles (alcohol use and depression as mental illness). Nevertheless, the authors included other factors such as distance from the clinic, which, as mentioned, is not relevant in Spain.

Moreover, our score showed the same specificity (86% vs. 87%) and higher sensitivity (87% vs. 59%).^15^ In another study published in 2017, Wohl et al. aimed to create and test an instrument to estimate future adherence to and persistence with ART. They used two scale development approaches: a 10-item survey (which took ∼5 min to complete) and a 30-item survey (10 min).^16^ The disadvantage, compared with our score, lies in the need for patient collaboration.

Furthermore, this tool can only be applied if ART has been started, whereas ours is conceived to be applied in ART-naïve patients. Finally, in 2021, Chen et al. developed a predictive model for ART adherence based on data from the Chinese population.^26^ One of the predictors included in this model was side effects. As in previous studies, this tool is only applicable to patients who have already started ART.

The importance of this study lies in the ability to not only identify patients at high risk for nonadherence but also detect other problems related to the patient and act consequently by referring them, for example, to social services or to drug cessation programs. Given that the cultural or idiomatic barrier risk factor was also statistically significant in the final model, increased investment in cultural mediators could improve adherence in the future.

In addition, the number of outpatients in our hospitals has grown considerably in recent years. We expect the same trend to continue in the future. It is necessary to optimize resources and change the current health system model, focused on drug efficiency, to another focused on patient adherence. In this context, the Strategic Map for Pharmaceutical Care for Outpatients (MAPEX, Spanish acronym) was born in Spain. This project led by the pharmacist, R. Morillo, aims to empower the patient. For this, the use of new information and communication technologies, real-world data, and big data will be essential.^27^

With fast and easy application of our score, patients are classified according to the risk of nonadherence and it is possible to optimize resources by establishing different assistance models. For example, providing 3 or 6 months of ART to patients at no risk of nonadherence could be particularly beneficial in the context of COVID-19. In addition, high-risk patients may then be prioritized for close clinical monitoring and prioritized treatments that have shown better adherence, such as STR.

This study has several limitations. First, it is a retrospective study and data were extracted from medical records. This was possible because in the Hospital de Mar, both doctors and pharmacists interview naïve patients and collect the variables used in this study. However, the fact that there was no standardized questionnaire to collect the information constitutes a bias in the study. Risk factors were collected only at the start of the study and therefore our model cannot account for variations over time.

If patients were nonadherent at one time, they were considered nonadherent forever. Moreover, the follow-up period was different between patients. In the future, it will be important to perform a prospective study to reassess the risk factors for nonadherence and the adherence status over time and to restrict the follow-up for all patients to a common time frame.

Second, the derivation and validation cohorts included PLHIV from a single center, thus inducing a selection bias. The proportion of drug users with a cultural barrier and LTFU was higher in the validation cohort due to the dynamics of the population that was treated at our center, a fact that should be taken into account when validating the results. Since these patients are not fully representative of the whole population of PLHIV, external validation will be critical to demonstrate the generality and utility of this risk score.

Third, the model was validated using split-sample validation and this method is less efficient than cross-validation.^28^ Fourth, the definition for heavy alcohol use was the same for men and women (>14 drinks/week), but the NIAAA recommends a different cutoff point for women.^5^ However, this fact was considered less relevant as 85% of the included patients were men.

Fifth, the variable, prejudgment by patient, did not include internal stigma and anticipated stigma, factors that increase the risk of poor ART adherence.^11^ Sixth, the ART regimen was not included in the multivariate analysis. Many studies have shown that the amount of pills^13^ and the treatment with protease inhibitors^3^ are risk factors for nonadherence. However, the proposed score was developed to be applied before ART initiation, and the results were used to select the most appropriate treatment regimens for each patient.

Finally, pharmacy refills in the hospital and patient self-reports were the methods used to assess adherence, but the limitations of these methods for estimating adherence are widely known.^29^

## Conclusions

The achievement of optimal adherence to ART is essential to ensure treatment efficacy and to minimize the emergence of resistance to ART and viral failure. This validated predictive score may be a useful tool to identify patients at high risk for nonadherence to ART and with high sensitivity, specificity, and negative predictive values.

Due to its simplicity, this score could be easily applied to all patients in daily clinical practice and also in future ART adherence studies. This predictive tool will allow stratification of patients to optimize resources by establishing different strategies for pharmacologic and therapeutic follow-up.

## Appendix

**Appendix Table A1.** Example of Total Score and Nonadherence Probability for Two Subjects

	Beta coefficient, βj	Score (over 100), βsj	Values, xj (0 = no and 1 = yes)	
			Subject 1	Subject 2
(Intercept)	−2.739	—	—	
Alcohol use	1.710	15	1	1
Substance abuse	0.899	8	1	0
Cultural or idiomatic barrier	2.007	18	0	0
Unstable housing	1.135	10	0	1
Prejudgment by patient	2.401	21	0	0
Previous appointment failures	2.352	21	1	1
Severe mental illness	0.743	7	1	0
Total score, TS = Σβsjxj	—	—	51	46
Linear predictor, LP = Σβjxj	—	—	2.965	2.458
Nonadherence probability, *p* = (1 + exp − LP) −1			95.1%	92.1%

TS for subject 1: 15^*^1 + 8^*^1 + 18^*^0 + 10^*^0 + 21^*^0 + 21^*^1 + 7^*^1 = 51.

TS for subject 2: 15^*^1 + 18^*^0 + 18^*^0 + 10 + 1 + 21^*^0 + 21^*^1 + 7^*^0 = 46.

LP for subject 1: −2.739 + 1.710^*^1 + 0.899^*^1 + 2.007^*^0 + 1.135^*^0 + 2.401^*^0 + 2.352^*^1 + 0.743^*^1 = 2.965.

LP for subject 2: −2.739 + 1.710^*^1 + 0.899^*^0 + 2.007^*^0 + 1.135^*^1 + 2.401^*^0 + 2.352^*^1 + 0.743^*^0 = 2.458.

*p* for subject 1 = (1 + e − 2.965) − 1 = 0.951 = 95.1%.

*p* for subject 2 = (1 + e − 2.458) − 1 = 0.921 = 92.1%.
